# Large-Scale Transcriptome Analysis of Retroelements in the Migratory Locust, *Locusta migratoria*

**DOI:** 10.1371/journal.pone.0040532

**Published:** 2012-07-06

**Authors:** Feng Jiang, Meiling Yang, Wei Guo, Xianhui Wang, Le Kang

**Affiliations:** 1 State Key Laboratory of Integrated Management of Pest Insects and Rodents, Institute of Zoology, Chinese Academy of Sciences, Beijing, China; 2 College of Life Science and Technology, Shanxi University, Taiyuan, Shanxi, China; University of Nebraska Medical Center, United States of America

## Abstract

**Background:**

Retroelements can successfully colonize eukaryotic genome through RNA-mediated transposition, and are considered to be some of the major mediators of genome size. The migratory locust *Locusta migratoria* is an insect with a large genome size, and its genome is probably subject to the proliferation of retroelements. An analysis of deep-sequencing transcriptome data will elucidate the structure, diversity and expression characteristics of retroelements.

**Results:**

We performed a *de novo* assembly from deep sequencing RNA-seq data and identified 105 retroelements in the locust transcriptome. Phylogenetic analysis of reverse transcriptase sequences revealed 1 *copia*, 1 BEL, 8 *gypsy* and 23 non-long terminal repeat (LTR) retroelements in the locust transcriptome. A novel approach was developed to identify full-length LTR retroelements. A total of 5 full-length LTR retroelements and 2 full-length non-LTR retroelements that contained complete structures for retrotransposition were identified. Structural analysis indicated that all these retroelements may have been activated or deprived of retrotransposition activities very recently. Expression profiling analysis revealed that the retroelements exhibited a unique expression pattern at the egg stage and showed differential expression profiles between the solitarious and gregarious phases at the fifth instar and adult stage.

**Conclusion:**

We hereby present the first *de novo* transcriptome analysis of retroelements in a species whose genome is not available. This work contributes to a comprehensive understanding of the landscape of retroelements in the locust transcriptome. More importantly, the results reveal that non-LTR retroelements are abundant and diverse in the locust transcriptome.

## Introduction

In insects, genome sizes vary across two orders of magnitude, i.e., from less than 100 megabases to larger than 10 gigabases (Gb) [1]. Genome sizes have fluctuated with periods of genome inflation via transposon activity throughout genome evolution. Hence, variations in genome size are mainly attributable to changes in the amount of transposable elements (TEs) [2]. Emerging genomic data from genome sequencing projects in different organisms show that TEs constitute a large portion of eukaryotic genomes (3%–45% in metazoans) [3]. These elements appear to have very similar structures, basically containing genes responsible for their transposition. TEs replicate in two main ways, involving either a DNA or an RNA intermediate, and they have been accordingly divided into two classes, namely: retrotransposons and transposons [4]. Retrotransposons are major contributors to genome size expansion by producing extra copies of retroelements from themselves throughout the genome via an RNA intermediate using a ‘copy and paste’ proliferation mechanism [5]. On the other hand, to attenuate genome size expansion via retroelement proliferation, inactive retroelements eventually decay and are excised from the genome under relaxed selection [6]. Thus, due to their ability to proliferate and their susceptibility to decay, retroelements appear to have a large impact on genome size variations [2]. In this context, retroelements are particularly important in determining genome architecture [2], [6], [7].

The abundance and evolutionary diversity of retroelements provide these important elements a tremendous potential as triggers of genome reshaping [8], [9]. Indeed, the dynamics of retroelements are now widely accepted as an important source of structural variations and genomic innovations. Their key roles in the evolution of genome architecture and gene regulation have also been recognised [5], [9], [10]. Retroelements have contributed substantially to genome size differences, with the larger genome being comprised of a diverse collection of retroelements in insects [11]. The majority of new retroelement copies produced by active retrotransposons are truncated and incapable of further retrotransposition. Due to the loss of retrotransposition function, the great majority of retroelements have suffered extensive mutations under a neutral rate. For this reason, their divergence to their original active retrotransposon corresponds approximately to the time elapsed since their arrival, such that ancestral families of retroelements are more divergent than younger ones [12]. These divergent retroelements have been accumulated in the genome gradually over a long period of time. Consequently, the non-prompt removal of retroelements could lead to genomic obesity by retroelement accumulation [13]. As a consequence of retroelement propagation, larger genomes may maintain a higher abundance and diversity of retroelements than smaller genomes [2], [11], [14]. The migratory locust, *Locusta migratoria*, has a genome size of 6.35 pg, which is twice the size of the human genome, or 36 times that of *Drosophila melanogaster* [1]. The larger size of the *L. migratoria* genome relative to other insects is believed to be directly related to the abundance and diversity of retroelements [15]. Benefiting from the well-established classification system of TEs, phylogenetic analysis based on reverse transcriptase (RVT) domains have been widely used to identify retroelements and reveal their genetic diversity [3], [16]. Therefore, determining the number and kind of retroelements colonising the locust genome is of tremendous significance and deserves great attention to gain insights into the genomic architecture of large genomes.

Numerous studies have been conducted to identify retroelements in genomic sequences. Our comprehensive knowledge on insect retroelements is limited to a few sequenced species – mainly organisms such as holometabolous insects (e.g., the fruitfly *D. melanogaster*, the beetle *Tribolium castaneum* and the mosquito *Anopheles gambiae*) [17], [18]. In spite of the substantially reduced cost of DNA sequencing by several orders of magnitude, genome sequencing is still labour intensive and time consuming [19]. In contrast to retroelements in genomic sequences, much less is known about the transcriptional landscape of retroelements. Although the integration of all transcribed retroelements into the host genome is not sufficient, transcription is the first and most essential step in the retroelement replicative cycle [9]. The transcribed retroelements are then reverse transcribed by the self-encoded RVT to integrate into the host genomes via an autonomous or non-autonomous mechanism [8]. Due to the accumulated mutation and truncation, most retroelements in their host genome are incapable of transposition into a new location. However, the transcription initiation of these decayed retroelements is still activated by their still-intact promoter in the retroelement fragments or the promoter from nearby protein genes [8]. Therefore, the transcriptional activities of retroelements can be detected not only by transcriptional initiation from active copies, but also by co-transcription of adjacent protein-coding genes alternatively [8]. With rare exceptions, the transcriptional activities of retroelements have been detected in all analysed eukaryotic transcriptomes, consistent with the recent finding that a substantial portion of retroelements contributes to the transcriptome [9], [20]. Thus, we set out to determine whether transcriptome analysis based on deep-sequencing strategies can be an efficient way of identifying and quantifying retroelements in *L. migratoria*, a non-model species with a large genome size.

The identification and quantification of retroelements in transcriptomes pose great challenges in assembly completeness (integrity of each retroelement transcript) and accuracy, due to the low throughput and generally not quantitative nature of large-scale expressed sequence tag (EST) sequencing [21]. Compared with the traditional polymerase chain reaction (PCR)-based or EST-based strategy, the cost-efficient transcriptome analysis based on deep sequencing strategies presents an efficient way for the high-throughput discovery of retroelements. It offers considerable advantages over the traditional methods, such as adequate coverage of assemblies, sensitivity for retroelements expressed at low levels, and high accuracy for quantifying expression levels [21], [22]. Hence, the identification and quantification of transcripts originating from retroelements may offer a potential indicator for the transcriptional activity of these elements. In combination with the unprecedented amount of high-throughput sequencing data, the identification and quantification of retroelements in transcriptome data offer an efficient alternative strategy in genome biology.

Increasing evidence demonstrates that retroelement have profound impacts on many different aspects of eukaryotic development [23], [24], [25]. Indeed, their contribution to transcriptomes has been acknowledged recently [9], [26]. The expression of only a few retroelements during development has been described in a limited number of species [27], [28]. Although transcripts for retroelements have been detected in several transcriptome studies, there is no study on how their expression changes on a genome-wide scale during development.

Phenotypic plasticity, the capacity of a given genotype to exhibit variable phenotypes in response to changing external conditions, is common in insects [29]. The effects of the external environment on the phenotype disrupt retroelement silencing, resulting in retroelement reactivation and altered retroelement expression [30], [31]. Changes in retroelement expression can rapidly reshape genome architecture by causing insertion, exon shuffling, chromosomal breakage, ectopic recombination and genome rearrangement [30]. They can also influence gene expression patterns by inserting into promoters or enhancers, by providing binding sites for transcription factors, by regulating the chromatin conformation, or by governing splicing and polyadenylation patterns [8], [32], [33]. Given their involvement in the genetic causes of phenotypic plasticity, retroelements are considered to be some of the essential forces affecting genomic plasticity, thereby activating or inhibiting gene expression [32], [34], [35], [36]. The migratory locust exhibits extreme phenotypic plasticity, transforming between a cryptically coloured, solitarious phase and a conspicuously coloured, gregarious phase [37]. Solitarious and gregarious locusts differ in many phenotypic traits, including colour, shape, metabolic physiology, hormonal regulation and immune response, collectively termed “phase-related traits” [38]. Altered environment conditions involved in swarm formation and mass movement are primarily responsible for phenotypic changes in response to increased population density [39], [40]. Under high population density, gregarious locusts form large, migrating swarms that can cause substantial damage to pastures and crops.

With the recognition that retroelements account for the genome size expansion in both animals and plants, retroelements can be speculated to constitute a large fraction of the locust genome and have a great influence on its genome evolution [5], [7], [31]. Despite the finding that a substantial portion of retroelement-related transcripts are expressed in the small RNA and the EST data in our previous studies [15], [41], the features of retroelements in locusts are still poorly understood. Systematic studies that characterise these elements are also not yet available. To facilitate the discovery and identification of retroelements in locusts, we present the first *de novo* transcriptome analysis for retroelements based on deep sequencing approaches. The *L. migratoria* transcriptome offers a great opportunity for determining whether retroelements are indeed more abundant and diverse in the locust genome. It also allows us to facilitate a comprehensive investigation of the transcriptional activities of retroelements, considering that they are assumed to play important roles during development and in phenotypic plasticity.

## Results

### Consensus sequence reconstruction of retroelements

A total of 447 million reads from deep sequencing libraries in the fourth instar stage were used for assembly by the Multiple-*k* strategy. All contigs from the different k-mer assemblies were then pooled together for a final assembly. The scaffolds inferred from the paired-end information were verified by translation mapping methods, which search orthologous regions in the protein sequences from Repbase using translated contigs. Transcripts longer than 300 bp were retained for further homology searches to ensure the accuracy of retroelement identification. Given that the structure signatures are almost absent in the transcriptome, retroelements were sought by an approach that relied on homology searching of the known retrotransposon proteins using the protein-based RepeatMasking program to identify transcripts that contain an inner region of retrotransposon homologous proteins. In an attempt to avoid false-positive identification, the following stringent criteria were used: (1) its protein length was at least 200 amino acids and the *E* value was less than 1*E*-25, and (2) its sequence did not show homology with the functional proteins from non-redundant database of NCBI, except for retrotransposon proteins. Transcripts showing homology with transposases were classified as DNA TEs and thus were removed from further analysis. To distinguish the consensus sequences produced by transcriptome data from those obtained by genome-scale analysis, we defined the “retroelement,” instead of the “family,” to the consensus sequence reconstructed in this study. Thus, the retroelement was assigned based on the following criterion: two transcripts belong to the same retroelement if they share 80% (or more) sequence identity over 80% of their sequences [3]. The resulting assembly formed 105 distinct retroelements with a maximum length of 8,863 bp. Ten retroelements were randomly selected for RT-PCR validation, and nine of them were successfully amplified and sequenced by cloning into pGEM-T vector (Table S1). Our retroelement dataset also included LmI retroelement, which was recently cloned by a PCR-based method using the SMART RACE technology [15]. Probably due to the intrinsic nature of retroelements, the majority (73%, 77/105) of transcripts ranged from 500 bp to 3,000 bp in length and transcripts longer than 3,000 bp were under-represented (27%, 28/105, Figure S1). We analysed the protein domain organisation of the 105 retroelement transcripts by both HMMER and protein-based RepeatMasking searches. Although the searches failed to detect the sequential domain structures, i.e. PRO-RVT-RNH-INT in *gypsy* (PRO, protease; INT, integrase; RNH, ribonuclease H), in a large portion of retroelement transcripts, the structures could still be categorised according to the homologies of their *pol* or *gag* polyproteins.

### Identification of full-length retroelements in the locust transcriptome

**Figure 1:**
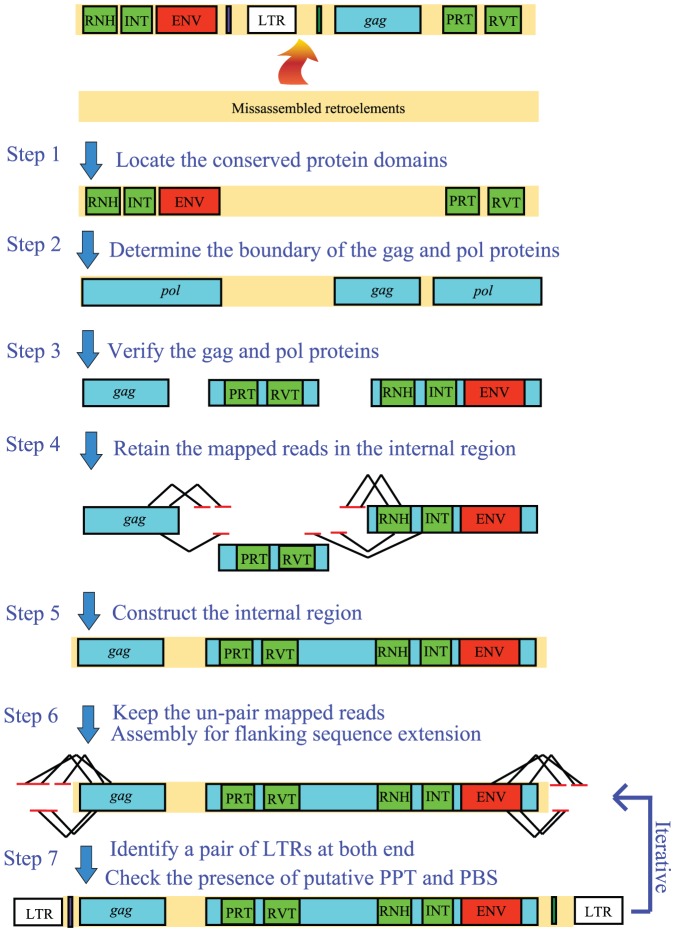
Strategy for the identification and characterization of full-length LTR retroelements in the *L. migratoria* transcriptome. Rectangles indicate protein domains. The polylines in step 4 and 6 indicate the paired-end reads. The misassembled LTR retroelement is presented at the top of the figure.

The definition of full-length long terminal repeat (LTR) retroelements is limited to those that contain a functional *gag*-*pol* structure, which is essential for providing the transposition-required structural components and enzymatic activities. Full-length LTR retroelements were identified based on multiple structural rules: detection of a pair of similar LTRs at both ends, presence of putative *gag* or *pol* open reading frames (ORFs), internal domain structure, polypurine tract sites (PPT) and primer binding sites (PBS) at the flanking end of LTRs. A novel strategy based on an iterative assembly process was adopted to reconstruct the complete region of retroelements. A schematic representation of our assembly strategy is shown in Figure 1. In total, five transcripts, designated as *Soty*, *Beri*, *Boyu*, *Kokol* and *Wusur*, were identified as LTR retroelements, as described below.

We performed a structural analysis of the full-length LTR retroelements. The domains identified in ORFs of these retroelements are presented in Figure S2. Structural analysis revealed that the main feature of known LTR retroelements was present in each full-length LTR retroelement. For each element, we also determined the PPT, PBS, and LTR length. In the absence of a previous reported dataset of tRNA gene sequences in *L. migratoria*, the PBS region was determined by the comparison of a comprehensive collection of insect tRNAs. In general, the two conserved sites, PPT and PBS, were located in the internal region of each LTR retrotransposon. To identify the PBS sequences, similarity searching against tRNA datasets was performed in the adjacent region of its LTR sequences. The PBS in *Kokol*, *Boyu* and *Wusur* corresponded to *tRNA-Ser*, *tRNA-Met* and *tRNA-Val*, respectively. *Kokol* and *Boyu* have a predicted PPT located immediately upstream of the right LTR. However, no clear PPT can be assigned to *Wusur*.

Two full-length retroelements were also identified as non-LTR retroelements, named *Rter* and *Limi*. *Rter* is a LINE/RTE retroelement, a clade of retroelements widely distributed in animals [42]. It possesses a 967-amino-acid ORF with RVT and endonuclease domains in positions similar to those of EXPANDER1. It also contains a short 3′ UTR composed of AT-rich nucleotides, a characteristic consistent with other members of the RTE clade. The RVT domain belongs to a family of RNA-dependent DNA polymerases that reverse-transcribes single-stranded RNA into double-stranded DNA (PF00078, RVT family). The endonuclease domain belongs to a large family of proteins, including magnesium-dependent endonucleases and phosphatases involved in intracellular signalling (PF03372, endonuclease/exonuclease/phosphatase family). At the 3′ end, we found a poly-A tail, indicating that *Rter* is a fully processed transcript of RNA polymerase II. For *Limi*, the protein-based RepeatMasking analysis of the coding regions showed significant sequence similarities to the nimbus from *Schistosoma manson* [43], suggesting that *Limi* belonged to the a newly defined clade, Nimb. This clade has been recently recognised to be an independent clade, which included members from insects, molluscs, and fishes [44], [45]. In nucleic acid binding proteins (also known as ORF1), three cysteine-rich motifs that form zinc finger domains of the CCHC type were detected in all three full-length retroelements : CX_2_CX_4_HX_4_C type, CYQCHRFNHTSQSC; CX_2_CX_3_HX_4_C type, CVTCGKEAHEGIC; CX_2_CX_3_HX_6_C type, CINCNGNHAASSREC. Apart from the RVT domain and AP (apurinic) endonuclease domain, the ORF2 of *Limi* also encoded an RNase H domain, which is responsible for the degradation of DNA/RNA hybrids. A poly-A tail could also be found located at the 3′ end of its UTR.

### Distribution of retroelements in different clades

**Figure 2:**
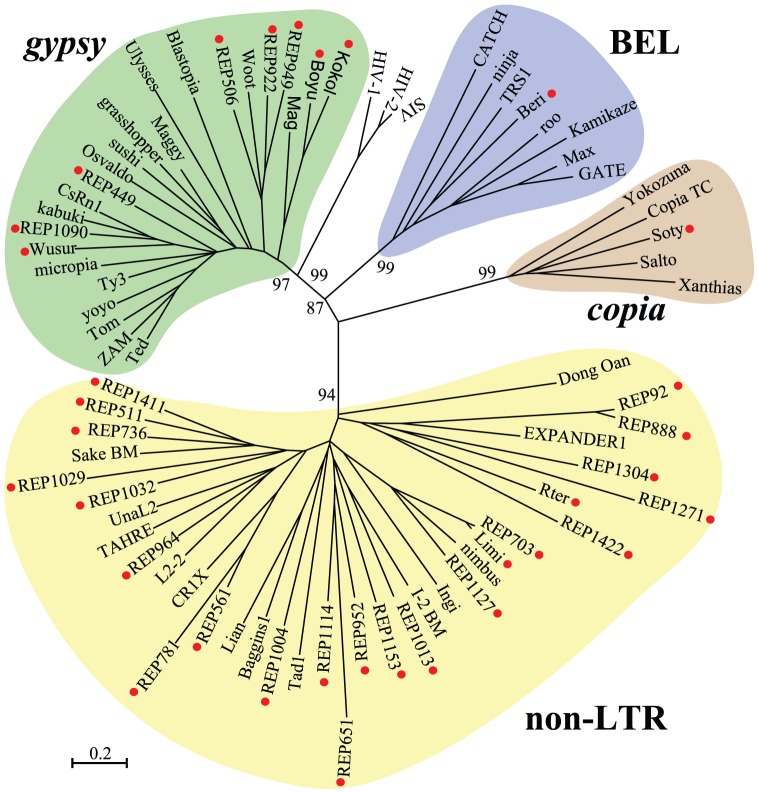
Global phylogenetic trees using all identified retroelements of reverse transcriptase sequences in the *L. migratoria* transcriptome. The red circles indicate the retroelement identified in this study. The names of elements from previously described retrotransposons are given.

To understand the phylogenetic inference of locust retroelements, the phylogeny based on the RVT sequences was analysed in the context of representative members of known clades. A total of 33 retroelements were selected because they showed sequence similarities to RVT sequences by HMMER searches (Figure 2). The potential RVT coding region of the remaining retroelements was absent or fragmented, although at least part of their sequences showed recognisable regions of proteins encoded by known retrotransposons. These 33 locust retroelements represented divergent lineages in previously established clades. The representation of transcribed retroelements appeared to be biased towards the non-LTR and *gypsy* clade, and only two retroelements corresponding to the *copia* or BEL/Pao clade were identified. In terms of the number of divergent retroelements in a clade, the rich diversity of the non-LTR and *gypsy* clade was observed. For example, six retroelements were grouped together with EXPANDER1, a member of RTE clades firstly identified in the genome of zebrafish.

**Figure 3:**
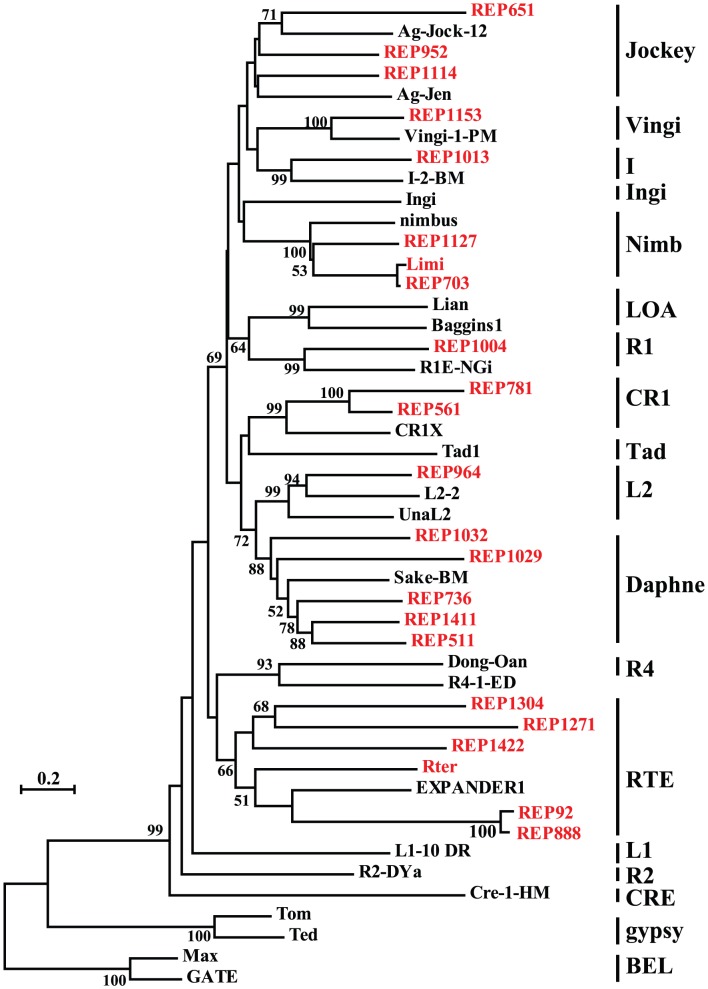
Phylogenetic relationships among the 12 well-established clades of non-LTR retrotransposons. Three newly identified clades, Vingi, Nimb and Daphne, were also included. This tree was constructed from the reverse transcriptase sequences using the neighbour-joining method. Bootstrap values less than 50 are not shown. The known retrotransposons from other species were retrieved from the GenBank and Repbase databases. The sequences in red indicate the non-LTR retroelements identified in this study.

Non-LTR retrotransposons constitute a diverse group of elements that are classified into 12 well-established clades [12]. In total, 23 retroelements were classified as non-LTR retroelements, accounting for 70% (23/33) of all identified non-LTR retroelements. Members from 6 of the 12 non-LTR clades were detected, namely, CR1, L2, I, R1, Jockey and RTE (Figure 3). Members from three newly identified clades, Vingi, Nimb and Daphne, were also detected [16], [44], [46]. These clades remarkably differ in the diversity of members. The majority of non-LTR retroelements corresponded to the two clades, RTE clade and Daphne clade. Among the nine clades that occupy the *L. migratoria* transcriptome, a large group of RTE clades was evident, which is consistent with a previous analysis in insects [47]. RVT-phylogeny analysis revealed the presence of six distinct retroelements, indicating that RTE is the most abundant and diversified clade of non-LTR retroelements in the *L. migratoria* transcriptome. Another diversified lineage, consisting of five distinct retroelements, was also detected in the Daphne clade.

**Figure 4:**
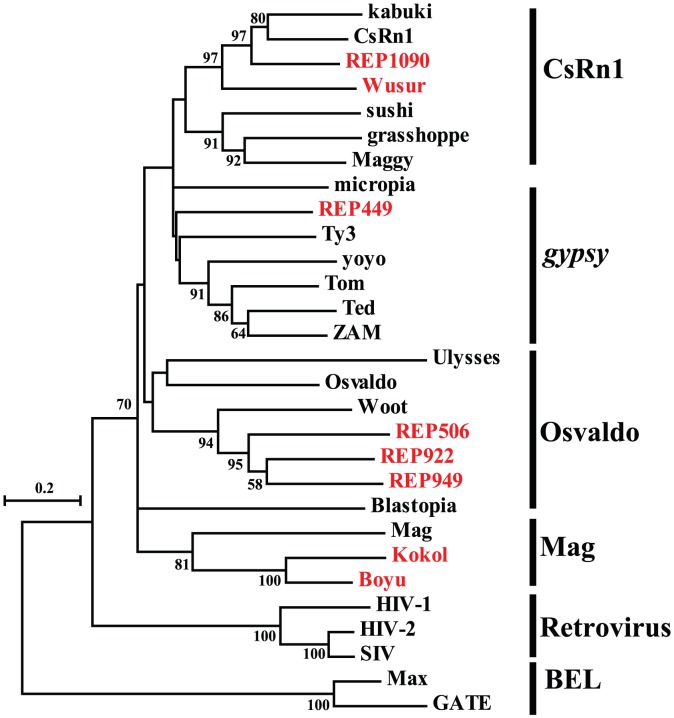
Phylogenetic relationships of the *gypsy* retrotransposons. Phylogenetic relationships were inferred by the neighbour-joining method. This tree was constructed from the reverse transcriptase sequences using the neighbour-joining method. Bootstrap values of more than 50 are shown in the branches. The known retrotransposons from other species were retrieved from the GenBank and Repbase databases. The sequences in red indicate the *gypsy* retroelements identified in this study.

Among the four main clades (*gypsy*, BEL, *copia* and DIRS), the most abundant LTR clade in insect genomes is *gypsy* [48]. The *gypsy* clade represents an extraordinary diversity of lineage distribution in different insects, and is categorised into six main lineages, namely, *gypsy*, Osvaldo, Mag, CsRN1, Mdg1 and Mdg3 [26], [49]. In particular, the *gypsy* retrotransposons in the *A. gambiae* genome belong to five distinctive lineages, whereas the retrotransposons in the *Daphnia*. *pulex* genome belong to two main lineages [48]. A total of eight retroelements were identified in the *L. migratoria* transcriptome, and they are split into four lineages that exhibit higher diversity than in *A. gambiae* and *D. pulex*. All the *gypsy* retroelements fall into several known lineages as described for the *gypsy* clade. Given that *gypsy* retrotransposons have a domain order of PRO-RVT-RNH-INT, the relative position of RVT and INT domains in the *pol* polyproteins also supported our classification of RVT-phylogenies. The neighbour-joining tree reveals that two retroelements, *Boyu* and *Kokol*, are clustered closely with the Mag lineage to represent a distant lineage from other *gypsy* lineages (Figure 4). Among the six retroelements in another macro-lineage, a relatively diversified lineage, supported with high bootstrap values, can be clearly distinguished: a lineage closer to the Woot element of *T. castaneum* from the Osvaldo lineage. In addition, REP1090, a retroelement that is closest to the CsRN1 retrotransposon, is grouped with *Wusur*.

Only one member of *copia* retroelements, *Soty*, could be identified in our RVT phylogeny analysis, following the trend of reduced *copia* abundance in animal genomes. The closest member from the *copia* clade is Copia_TC of *T. castaneu*. BEL retroelements are also rare in the *L. migratoria* transcriptome, and only one BEL retroelement with an RVT domain could be found. Using an RVT of the *gypsy* element as an outgroup, we constructed the neighbour-joining tree based on their RVT similarities (not shown). In this tree, the BEL retroelement was close to the *TRS1* retrotransposon in the nematode *Trichinella spiralis*.

### Recent transpositional activity of retroelements

Newly inserted retroelements from an active copy in the genome are transcribed with the flanking transcriptionally active region. They are nearly identical with the active copy in sequences. Most of these newly arrived retroelements have been subject to selective constraints and are likely to be gradually degraded from the genome by deletions. If these retroelements were retrotransposed into their current region in recent evolutionary history, the time since their transposition is not sufficient to degrade these retroelements and complete transpositional features can still be identified. Hence, transpositional features including the presence of full-length copies, intact protein component and similar LTRs can provide clues to the recent transpositional history of a retroelement. Full-length copies and intact ORFs of LTR retroelements in *Soty*, *Beri*, *Boyu*, *Kokol* and *Wusur* retroelements indicate their recent amplification from an active element, without adequate time for divergence or removal by unequal homologous or illegitimate recombination at the transcribed region. Considering that the transposition of LTR retrotransposons depends on the a pair of LTRs at both 5′ and 3′ ends, the LTR sequences flanking intact LTR retrotransposons are identical when integrated at a new site. The LTR region similarities for the *Soty*, *Beri*, *Boyu*, *Kokol* and *Wusur* are 97%, 99%, 92%, 94% and 100%, respectively. The presence of identical or highly similar LTRs suggests that these LTR retroelements were transposed into the transcriptionally active region within a short evolutionary time and showed recent retrotranspositional activities.

The mechanism of LINEs in driving genome evolution is not well understood. Nevertheless, it is of great interest that active LINEs showing retrotranspositional activities can be transcribed into RNA and then reintegrated into their host genome. Both *Rter* and *Limi* contain an internal promoter, one or two ORFs, a 3′UTR and a poly-A tail. Retroelements show less repression from their host and can rapidly proliferate after invasion into an uninfected genome [50]. Consequently, a large number of full-length copies can be integrated into their host genome. Because the accumulation of retroelements is deleterious to their host, a solution to silence them will be developed by their host. Once the active retroelements are silenced, no new transposed copy will be produced. The already transposed copies will be degraded, and retroelement extinction could occur through the accumulation of mutations/deletions within transposed copies. With time passed by, full-length copies cannot be identified. Thus, the active retroelements that contains complete structures can be identified in the transcriptome. Otherwise, we could only identify fragmentary copies of retroelements that are not recent or currently active, because ancient transposed copies have been experienced mutations and deletions. The full-length copies of *Rter* and *Limi* are assembled in the transcriptome, indicating that they are actively transcribed from an activated source copy or have arrived at the transcriptionally active region recently [51], [52]. In either case, the presence of full-length copies in the transcriptome reinforces the idea that *Rter* and *Limi* have been activated or deprived of retrotranspositional activities very recently.

### Transcriptional activity of retroelements

**Figure 5:**
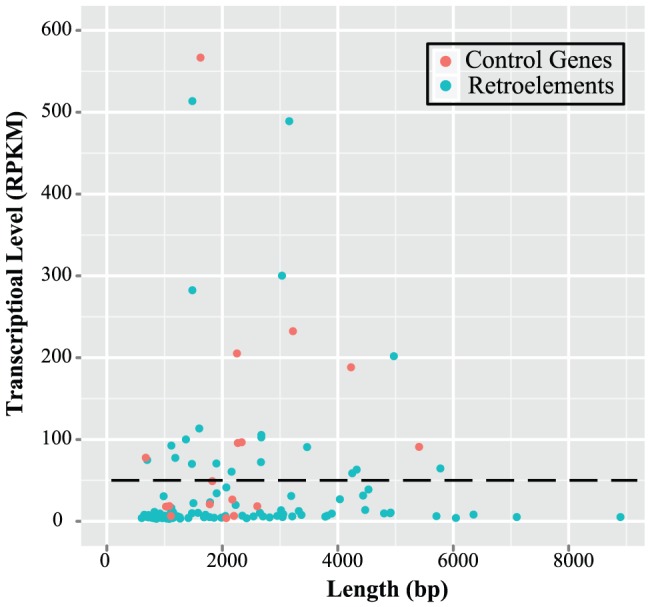
Transcriptional activities of retroelements in the fourth instar nymphs of solitarious locusts. The x-axis indicates the length of sequences, and the y axis indicates the transcriptional level in RPKM values. Due to its highly transcriptional activity, elongation factor 1 alpha is omitted in this figure.

To assess the level of transcriptional activities of retroelements, we analysed the deep sequencing RNA-seq results in the fourth instar nymphs of solitarious locusts. We compared their transcriptional level to other control gene sets, which comprised 18 house-keeping genes, including Elongation factor 1 alpha, Actin-5C, Tyrosine 3-monooxygenase and Glyceraldehyde-3P-dehydrogenase (Table S2). The 105 retroelements expressed in the fourth instar stage had reads per kilobase of the transcript per million mapped reads (RPKM) values from 0.5 to 511. Most of these retroelements showed low (0.5–10 RPKM, 68%, 71/105) to moderate (10–50 RPKM, 13%, 14/105) expression (Figure 5). The expression levels of 10 retroelements were validated by qRT-PCR experiments to verify our RNA-seq data (Figure S3). Based on the homology to *gag* or *pol* proteins of known retrotransposons, 20 highly expressed retroelements (above 50 RPKM, Table 1), were classified into 1 BEL, 4 *gypsy* and 15 non-LTR retroelements. All the values in RPKM fell into the range observed in the control gene sets, and no extremity was detected. Various copies of each retroelement family with high sequences similarities are widely distributed in the genome. Thus, the transcriptional activities of a specific transcript in an exact locus are often not feasible to determine. Therefore, it should be noted that the transcriptional activities measured here for each retroelement may be the contribution of copies in different loci at the transcriptionally active region. In other words, considering that the transcriptional activity for each retroelement was quantified from numerous copies, the expression level of each copy in different loci may be markedly lower.

**Table 1:** Highly expressed retroelements in fourth instar nymphs of solitarious locusts.

ID	RPKM	Length	Homologues	Clade	Superfamily
REP1361	56.32	4217	Vingi-1_Pp_1p	Vingi	Non-LTR
REP1124	69.9	2636	Vingi-1_BF_1p	Vingi	Non-LTR
REP1422	103.12	2642	RTE-1_NVi_1p	RTE	Non-LTR
REP885	511.14	1448	RTE-10_BF_1p	RTE	Non-LTR
REP1247	64.83	3311	RTAg4_1p	R1	Non-LTR
REP955	60.72	4294	Penelope-5_NV_1p	Penelope	Non-LTR
REP1357	199.37	4938	Penelope-5_NV_1p	Penelope	Non-LTR
REP1274	297.76	3000	Nimb-2_CQ_2p	Nimb	Non-LTR
REP1054	62.18	5744	I-2_DR_2p	I	Non-LTR
REP1379	90.14	1084	I-3_DR_1p	I	Non-LTR
REP414	111.04	1567	I-2_DR_2p	I	Non-LTR
REP1094	486.6	3126	I-2_DR_2p	I	Non-LTR
REP1024	58.2	2127	CR1-20_NV_1p	CR2	Non-LTR
REP561	279.99	1448	CR1-20_NV_1p	CR2	Non-LTR
REP562	67.73	1440	CR1-1_BF_2p	CR1	Non-LTR
REP942	88.32	3435	CR1-1_BF_2p	CR1	Non-LTR
REP449	68.25	1864	Gypsy-23_IS-I_2p	*gypsy*	LTR
REP563	72.52	665	Gypsy-9_DWil-I_1p	*gypsy*	LTR
REP587	75.13	1152	Gypsy-39_DPu-I_1p	*gypsy*	LTR
REP743	97.65	1336	Gypsy-9_DWil-I_1p	*gypsy*	LTR
REP1212	113.78	2181	Copia-4_XT-I_2p	*copia*	LTR
REP1039	207.94	1646	Copia-4_XT-I_2p	*copia*	LTR
REP764	389.32	1107	Copia-4_XT-I_2p	*copia*	LTR
REP702	73.64	777	BEL-30_CQ-I_1p	BEL	LTR
REP797	100.12	2642	BEL-30_CQ-I_1p	BEL	LTR
REP874	100.62	1268	BEL-30_CQ-I_1p	BEL	LTR
REP847	114.52	1268	BEL-30_CQ-I_1p	BEL	LTR
REP941	146.67	1168	BEL-30_CQ-I_1p	BEL	LTR
REP790	162.74	1004	BEL-30_CQ-I_1p	BEL	LTR

### Divergence distribution of retroelements in the locust transcriptome

**Figure 6:**
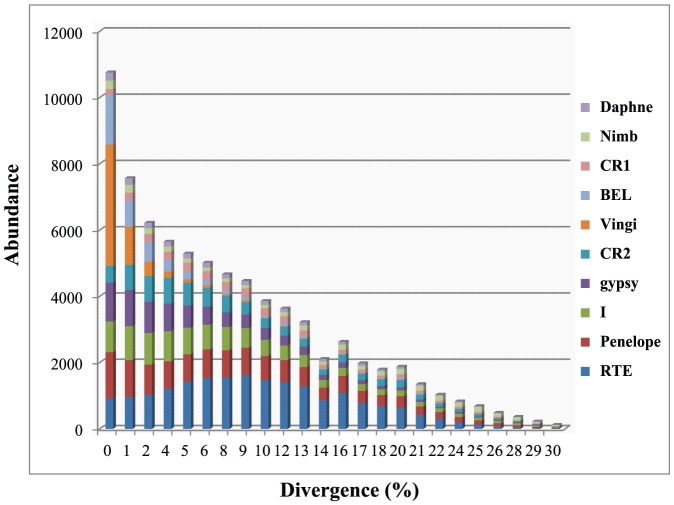
Transcriptional composition of the *L. migratoria* transcriptome. The values on the x-axis correspond to the divergence rates, and the values on the y-axis are the total transcriptional abundance.

To assess further the evolution of retroelement clades in the locust transcriptome, we conducted a more detailed analysis of the relationships between individuals within each clade. Sequences for individuals within each clade were collected and aligned separately, and pairwise divergences with the consensus copy were calculated. To avoid the potential artifacts arising from the complexity of retrotransposon assembly, we used the Solexa reads instead of the assembled transcripts to estimate the diversity of retroelements. This resulted in a divergence distribution that reflected the diversity of each clade and composition of transcriptome. Figure 6 shows that the locust transcriptome contains a substantially higher proportion of the RTE clade. Given that RTE is the most diversified clade in the locust transcriptome, the major contribution to this higher proportion may come from numerous RTE copies.

**Figure 7:**
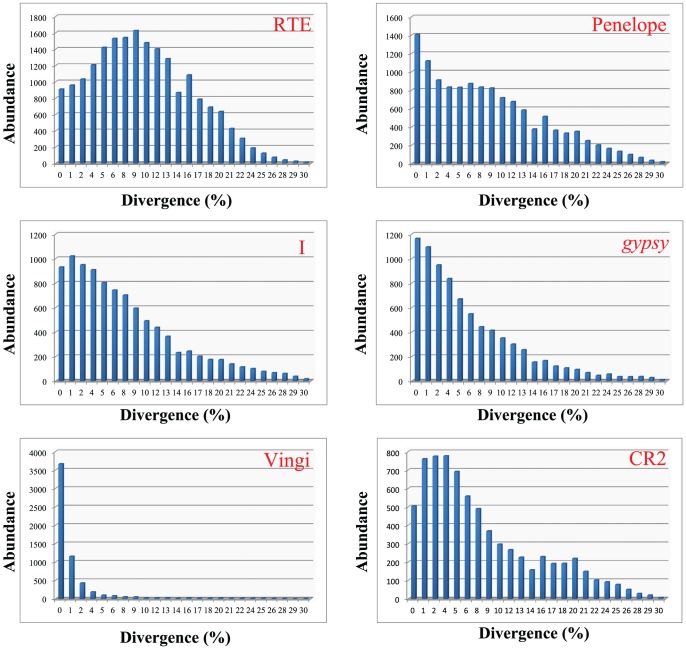
Pairwise divergence distribution of clades with a high transcriptional abundance. The values on the x-axis correspond to the divergence rates of the retroelements for each clade. The values on the y-axis are the transcriptional abundance.

Remarkably, most clades displayed numerous representations with relatively smaller divergence in the locust transcriptome (Figure 7). This scenario together with the gradually decreased representation from small divergences to large divergences strongly suggested that the retrotransposition events that occurred at the transcriptionally active region are occurring in the locust genome and many clades have arrived at the current locus for a long period. All the clades of non-LTR retroelements, except for the Vingi clade, covered a wide range of the divergence rates. The broader divergence distribution suggested that many copies of them exists in the transcriptionally active region and have not been eliminated by the selective sweep. The Vingi clade was an exception due to the narrow range of divergence rates. The scarcity of representations with large divergences indicated that the Vingi clade invaded the locust transcriptome recently, and it could be assumed that only a few highly similar copies of the Vingi clade may be present in the locust transcriptome.

The presence of a peak of abundance with 9% divergence was observed in the RTE clade, the most abundant and diversified clade in the locust transcriptome. Considering that the sampling of transcriptome data is biased, either a copy with extremely high transcriptional activity or many copies with low/moderate transcriptional activity could be expected to result in a high abundance in its clade. Therefore, the transcriptional level for each retroelement was assessed for the RTE clade. We detected an extremely highly expressed RTE retroelement, REP885 (Table 1), which is responsible for a dominant portion of transcriptional activity of the RTE clade. Therefore, many divergent copies with low/moderate transcriptional activities of REP885 lead to a peak of abundance for RTE clade, which is consistent with our findings from the RVT phylogeny analysis indicating that the RTE clade is a diversified lineage. Using 9% divergence rate as the calculation point to estimate the time of proliferation, we estimated that the latest burst of RTE clade was initiated around 2.8 million years ago.

### Developmental expression profiles of retroelements

**Figure 8:**
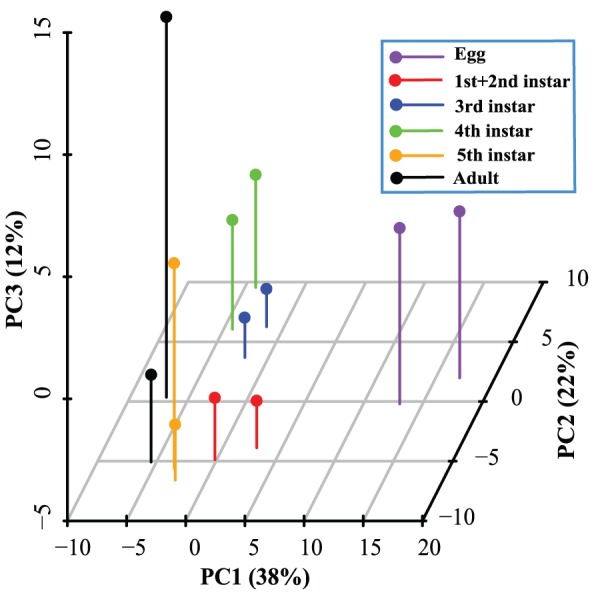
Principal component analysis of retroelements in the *L. migratoria* transcriptome. Two phases in the same developmental stage are plotted with the same colours.

Phase changes in the migratory locust represents a very attractive model system to investigate the mechanism underlying environment-dependent phenotypic plasticity [39]. Retroelement bursts and silences that occur in individuals who experience different environmental stresses during development result in genomic adaptations, possibly leading to a phenotypic diversity without being detrimental to the host [32]. Therefore, we performed a principal component analysis (PCA) on the expression profile data from the two phases of locusts in different developmental stages to visualise the transcriptional dynamics of retroelements. The first three principal components accounted for 72% of data variance. Interestingly, in the PCA plot, the PC1 axis, reflecting developmental variability, clearly separated the egg stage from the other stages, and accounted for 38% of the variance in the data set (Figure 8). Overall, the average RPKM values for the egg, first+second instar, third instar, fourth instar, fifth instar and adult stages, were 73, 44, 36, 33, 38 and 30, respectively (Figure S4). More than 75% (79 out of 105) of the retroelements had a larger value than the average value of different stages for each retroelement. Therefore, the high transcriptional activity of retroelements in the egg stage led to the obvious differences associated with the PC1 axis for the egg stage. The two phases at the same stage were always positioned together. The close relation in the PC1 axis and the progressive differentiation in the PC3 axis suggested that the distance along the PC3 axis reflected the differences between the two phases, because the PCA analysis was performed without any prior knowledge on the phase status or developmental stage. The fifth instar and adult stages exhibited distinct differences in phase traits [53]. The correlation of the PC2 axis to any developmental stages or biological function remains unclear.

## Discussion

Recent advances in genomics, particularly in high-throughput genome sequencing, have yielded comprehensive resources and information on the nature and structure of animal genomes. One of the major topics in genomics is the further elucidation of TEs, which contribute to a considerable portion of genomes. The C value (mass of DNA per haploid nucleus) is estimated to range from 5.28 to 6.35 for *L. migratoria*, indicating that its large genome size [1]. The large genome of *L. migratoria* can be presumed to be colonized by a substantial fraction of TEs, because the proliferation of retroelements leads to the expansion of genome size in both animals and plants [5], [7]. However, only more than 100 retroelements, 73 non-LTR retroelements and 32 LTR retroelements, can be identified in the locust transcriptome. These results suggested that the retroelements in a minority of loci contribute to the majority of the transcriptional activity in the locust transcriptome, although we cannot assess the transcriptional activity in each individual locus. To compare the abundance and diversity of retroelement clades among insects with different genome sizes, the data for *D. melanogaster* and *A. gambiae* were retrieved from Repbase and a previous study [54]. According to Repbase, there are 37 non-LTR families and 62 LTR families in the *D. melanogaster* genome. Obviously, the number of fruitfly non-LTR families showing transcriptional activity is much lower than that of locust non-LTR families, although the transcriptional landscape of non-LTR retroelements in fruitflies has not been described. In the *A. gambiae* genome, 22 non-LTR families and 30 LTR families, exhibit transcriptional activities [54]. The increased number of non-LTR retroelements demonstrates that in transcriptomes, non-LTR retroelements in locusts are more abundant than those in insects with a small genome size. For the purpose of comparison, the number of non-LTR clades showing transcriptional activity was also determined in *D. melanogaster* and *A. gambiae*. The non-LTR families in these two species are composed of five (Jockey, CR1, I, R1, and R2) and four (RTE, Jockey, I, and CR1) different clades, respectively. The nine non-LTR clades (Jockey, Vingi, I, Nimb, R1, CR1, L2, Daphne and RTE) identified in the *L. migratoria* transcriptome revealed that greater diversity of non-LTR retroelements in *L. migratoria* than in the other two insects, consistent with the increased number of non-LTR retroelements. Above all, non-LTR retroelements were indeed more abundant and diverse in the locust transcriptome. In view of the important roles of retroelements showing transcriptional activity in genome inflation, we propose that the non-LTR retroelement represent one of the main factors responsible for genome obesity in locusts.The evolutionary relationship of the Daphne clade with other non-LTR retroelement clades has been determined recently. They are assumed to be clustered together with the L2 clade, consistent with our phylogenetic tree [46]. Now, only two members of the Daphne clade, Sake_BM and Daphne-1_TCa, are identified in the genome of the insects, the silkworm *Bombyx mori*, and the beetle *T. castaneum*, respectively [45]. For the purpose of comparison, we used the deduced protein sequences of Sake_BM and Daphne-1_TCa in a TBLASTN search of retroelement family datasets in their genome sequences [18], and the transcriptional activities for the retroelement families were measured using the available expression data. Only one family of the Daphne clade was identified in both genomes, and the transcriptional activities were only detected in *B.mori*. Therefore, in terms of the number and diversity, the Daphne clade in the locust transcriptome represents a more successful group than that in the transcriptome of silkworm and beetle. Apart from the Daphne clade, the RTE clade is also a diversified lineage, and its member number is comparable to that of the Daphne clade. Considering the chances of retrotransposition events occurring in the transcriptionally active region, multiple members in both clades suggest that the locust genome is occupied by more retroelements from the Daphne and RTE clades than those from other clades.

Although a large number of novel retroelements can be identified in newly-available genomes, the proportion of retroelements that currently remains transcriptional currently is often unclear. Transcription represents the first step of retroelement transposition, even though only a portion of transcribed retroelements is successfully translocated into a new genomic location. Thus, an intact structure is not sufficient but necessary for active retroelements. The developments in genomic-scale analysis technology provide opportunities for quantifying gene transcriptional levels at an unprecedented depth and resolution, and can also be used to detect and quantify the transcriptional activity of retroelements. A previous study using tiling array data reported that eight intact retroelements in the *D. pulex* transcriptome [48]. In *A. gambiae*, five non-LTR families seem to exhibit signs of transcriptional activity [54]. In the present study, we identified two potential active non-LTR retroelements, showing a complete structure that contains the 5′UTR, one or two ORFs, a 3′UTR and a poly-A tail. We also identified at least seven non-LTR retroelements that included a complete homologous region of ORFs from known retrotransposon elements. Therefore, the number of intact non-LTR retroelements in the *L. migratoria* transcriptome is greater than that in the *D. pulex* and *A. gambiae* transcriptome. Taking into account the large genome size of locusts, these results are expected and they suggest that in insects, a larger genome size corresponds to a greater abundance of intact non-LTR retroelements in transcriptomes.

In the full-length LTR retroelements, the fact that two almost identical LTRs are present at both ends and the likelihood that two LTRs are assembled together into one set cause the full-length LTR retroelement to assemble into a wrong direction. In an attempt to solve this problem, we developed a novel approach based on both the similarities to the already described protein domains and the recognition of the intrinsic characteristics of retroelements, such as their domain orders and LTRs. Although our approach was successfully used to reconstruct the five full-length LTR retroelements in the transcriptome data, it was not feasible for identifying the ancient LTR retroelements flanked by divergent LTRs. Hence, our approach is more specialized for the detection of younger LTR retroelements. Overall, a total of 10 LTR retroelements, including 1 BEL, 1 *copia* and 8 *gypsy* retroelements, were identified in the RVT-phylogeny analysis. At least 56 and 12 families of intact elements exhibited transcriptional activities in the *D. pulex* and *D. melanogaster* genomes, respectively [48]. The LTR retroelement diversity of the *L. migratoria* transcriptome is only comparable with that of the *D. melanogaster* transcriptome, and is dramatically lower than that of the *D. pulex* transcriptome. Therefore, the proliferation activity of LTR retroelements is suppressed in the *L. migratoria* transcriptome, although we stress that the lower diversity of LTR retroelements may be artifacts caused by the complexity of retroelement assemblies in transcriptome data.

Our analysis of the divergence distribution of retroelements emphasizes a possible mechanism that could account for the larger size of the locust genome. We found that numerous divergent copies of retroelements accumulated in the locust transcriptome. The wide range of divergent rates indicates that retroelements do reach fixation in the transcriptionally active region of the locust genome. This divergent pattern is quite similar to that previously described in mammoth but not in anoles [12], [55]. The mammoth genome is 4.7 Gb, whereas the *Anolis* genome is only 2.2 Gb. In fact, mammoths represent a species with large genome sizes in vertebrates [55]. This finding suggests that large genomes are composed of numerous divergent copies of retroelements, implying that the *L. migratoria* genome has a slow turnover of retroelements. In *Drosophila*, the insertion of new copies is offset by the quick loss of older copies, leading to a rapid turnover of retroelements [56]. In contrast, the slow turnover of retroelements in the locust genome suggests that selection against retroelement maintenance has a small role in preventing retroelement accumulation. Hence, locusts may have a greatly reduced loss rate of retroelements. Although much less is known about the loss rate of retroelements in locusts, the abundance of divergent copies of retroelements indicates that the rate of retroelement loss may be lower in locusts than in other insects with a small genome size.

In the present study, the expression dynamics of retroelements during development revealed a relatively low level of retroelement silencing in the egg stage. DNA methylation represents an important mechanism for silencing retroelement transcription [30]. In insects, DNA methylation is almost absent, and thus plays little role in repressing the expression of retroelements [57]. However, an intermediate value for the amount of DNA methylation between *D. melanogaste*r and mammals has been proposed in *L. migratoria* recently [58]. A portion of methylated clones corresponding to retroelements indicate that silencing retroelement transcription by DNA methylation is also likely to be present in *L. migratoria*. Global DNA demethylation in mammals has been shown to play a key role during early embryonic development [57]. A previous study has shown that embryonic cells carry abundant L1 RNA, and L1 retrotransposition events are believed to occur mainly during embryogenesis [59]. Whether DNA demethylation in insects is similar to that in mammals during embryonic development is unknown [57]. We observed that retroelement expression was generally higher in the egg stage than in other stages. Our results further suggested that a higher expression is common in the egg stage for numerous retroelements, implying that the genome-wide demethylation after fertilization also relaxes the repression of retroelements in the egg stage in *L. migratoria* [60].

The reactivation of retroelement expression may be one of the possible ways of regulating phenotypic plasticity. The differentially expressed retroelements between the two phases of locusts are detected in the fifth instar and adult stages, when dramatic phenotypic traits appear [53], [61]. Retrotransposition in somatic tissues during development has been shown to lead to genomic plasticity within an individual, and is considered to play a potential role in phenotypic differentiation [22], [32]. Somatic retrotransposition in the neurogenic zones of the brain could occur in the nervous system [22]. These observations raise the possibility that retroelement activity may contribute to the intra-individual variation in genome architecture involved in phenotypic changes during neuronal development. The genes in the central nervous system are the key factors affecting the induction of phenotypic changes in *L. migratoria* [40], [61]. Thus, it remains an open question whether retroelement differences between the two phases at these two stages play a role in phase polyphenism. Methods for detecting structure variations for retroelement insertion/deletion are being gradually developed. Unfortunately, the *L. migratoria* genome has not yet been determined now. Therefore, it is of great interest to determine how the retrotransposition contributes to the non-heritable phase transition of locusts in future.

## Methods

### *De novo* transcriptome assembly

The raw sequencing reads were obtained from a recent study, which achieved a high coverage of the protein-coding gene content of the migratory locust by deep sequencing [53]. Transcriptome assembly using the Multiple-*k* method has been proven to improve substantially the assembly performance and increase the length of contigs [62], [63]. Given that only a single *k*-mer was used in the original study [53], an optimal overall assembly could not be yielded [64]. Therefore, we conducted multiple assemblies in this study using the Multiple-*k* method to improve the assembly and assist in retroelement reconstruction. Prior to assembly, we filtered out the reads where more than one-third of bases were ambiguous. We used the *de novo* assembler Trans-ABySS to assemble the deep sequencing paired-end reads of the fourth instar stage in *L. migratoria* [64], [65]. First, we conducted multiple assemblies by decreasing the *k*-mer length (k = 46, 41, 37, 33, 29, 25, 23, and 21). For each assembly, the reads used in the previous assembly were discarded, and a new assembly with a lower *k*-mer length was conducted with the remaining reads. Next, we pooled all contigs obtained from additional assemblies to form the final set of contigs. This approach allowed the inclusion of some redundant contigs. The program cd-hit and pairwise BLAT were used to map the final set of contigs against itself, and the redundancy was removed to produce the final assembly [66], [67]. Custom Perl scripts were used to retain the longest possible contigs.

### Retroelement Identification

To identify the retroelements in our assembled sequences, we downloaded all canonical retrotransposon protein sequences from Repbase and divided them into *gypsy*, BEL, *copia*, DIRS and Non_LTR classes [45]. Protein-based RepeatMasking (www.repeatmasker.org) searches of the assembled sequences were performed against the protein sequences from different classes to identify transcripts containing an inner homologous region of retrotransposon proteins. The presence of ORFs was translated in the same frame of nucleotide sequences using the NCBI ORF finder (www.ncbi.nlm.nih.gov/gorf). Retroelements can modify the non-retrotransposon protein-coding genes by inserting into the exon of the host gene or creating new internal exons [68], [69], and can thus be co-transcribed with the protein-coding genes. To remove the non-retrotransposon protein-coding exons, the non-retroelement ORF region with a length of greater than 300 bp was subjected to a BLAST search against the NCBI non-redundant database. All BLAST results were manually curated.

Profile Hidden Markov Models (PHMMs) provide a coherent theory for the probabilistic modeling of protein domain families, and are widely used to search for known domains in given protein references [70], [71]. To locate the positions of protein domains in polyproteins, PHMMs were generated from multiple Pfam alignments (PF00078, RVT_1; PF07727, RVT_2; PF00665, rve; PF00075, RNase_H; PF09668, Asp_protease; PF05380, Peptidase_A17; PF03372, Exo_endo_phos; PF03564, DUF1759; PF05585, DUF1758) of individual retrotransposon domains using HMMER [72]. Using these Pfam PHMMs, we then identified the domains in all retroelements by PHMM searches (*E*-value<0.0001). We obtained all identified domain sequences and aligned them within the same class using ClustalX [73]. Obvious errors of alignments were corrected, and boundaries of domains were manually verified.

### Retroelement identification

To distinguish the consensus sequences produced by the transcriptome data from those arising from genome-scale analysis, we defined the “retroelement,” instead of the “family,” to the consensus sequence reconstructed in this study. All the transcripts were grouped into clusters of retroelements sharing 80% or more in at least 80% of the aligned sequence. Transcripts belonging to the same retroelements are expected to be similar in sequences within these retroelements. Consensus sequences for each retroelements were obtained from the pair-wise sequence alignments using ClustalX [73]. To infer the consensus sequence, the most frequently appearing nucleotide in each position of sequences was retained by in-house Perl scripts independent of the occurrence number of retroelements covering that position.

### Sequence alignment and phylogenetic reconstruction

The phylogenetic tree that helped us categorize retroelements into known retrotransposon classes was constructed. Representative RVT sequences were downloaded from the NCBI GenBank database or Repbase: AAC28743, *yoyo*; CAA80824, *Tom*; AAA92249, *Ted*; CAA04050, *ZAM*; BAA92689, *kabuki*; AAK07486, *CsRn1*; AAO27306, *Ty3*; AAA21442, *grasshopper*; AAA33420, *Maggy*; AAC33526, *sushi*; CAB39733, *Osvaldo*; CAA39967, *Ulysses*; AAC47271, *Woot*; CAA32198, *micropia*; CAA81643, *Blastopia*; S08405, *Mag*; AAA76841, *HIV-2*; AAA47606, *SIV*; BAA74713, *Yokozuna*; XP969432, *Copia_TC*; NP057849, *HIV-1*; X92487, *Hsr1*; AAL55241, *Salto*; CAD32253, *Max*; BAD01590, *ninja*; CAA09069, *GATE*; AAN87269, *roo*; AB042120, *Kamikaze*; AAN15112, *CATCH*; EFV61807, *TRS1*; CACN01001643, *Tad1*; Repbase, EXPANDER1; Repbase, *Baggins1*; AAB65093, *Lian*; CAD21860, *Ingi*; Repbase, *Dong_Oan*; EF413180, *nimbus*; BAD72127, *UnaL2*; CAD65869, *TAHRE*; AAZ15237, *Kiri-22*; and XP001601755, *L2-2_NVi*. Multiple sequence alignments of RVT amino acid sequences were performed using ClustalW with default parameters [73]. Phylogenetic trees were generated by the Neighbor-joining algorithm, and the genetic distances were calculated by the Poisson correction model using the MEGA software [74]. Statistical support of the internal nodes was evaluated by bootstrap analysis with 1,000 replicates.

### Expression profiling

Prior to mapping the sequencing, reads with low quality bases and short lengths were removed. Mapping was carried out using the Burrows-Wheeler alignment (BWA) software [75]. The number of mapped reads for each retroelement was subjected to a scaling normalization to calculate the expression level in a unit of RPKM [76]. PCA was performed using the R software (http://www.r-project.org/).

### Divergence distribution and divergence time estimation

The abundance of various retroelements was estimated according to the following equation:
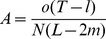
where *A* is the abundance of retroelements, *o* is the observed number of pairwise alignments, *T* is the transcriptome size, *l* is the length of sequencing reads, *N* is the total number of sequencing reads, *L* is the length of the retroelements, and *m* is the minimal length required to identify a sequence in a pairwise alignment. The divergence for each pairwise alignment was inferred from 75 bp Solexa reads using the RepeatMasker program (http://www.repeatmasker.org), and only the pairwise alignments with a length longer than 70 bp were used for the abundance estimation. Substitution rates in retroelements were used to estimate the divergence times, assuming that they are subject to decay with the same substitution rates under relaxed selective constraints. Therefore, the divergence time of each clade was estimated based on the one-parameter Jukes-Cantor model according to the following equation:
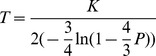
where *K* is the average number of substitutions per site and *P* is the sequence divergence rate. The average nucleotide substitution rate is set to 1.66×10^−8^ per site per year according to the substitution rates for a nuclear pseudo-gene in beetles [77].

### RT-PCR and qRT-PCR analysis

Total RNA was isolated using Trizol reagent (Invitrogen) according to the manufacturer's instructions. RNA quality was assessed using either agarose gel electrophoresis or a NanoDrop ND-1000 spectrophotometer. Total RNA was reverse-transcribed with oligo-dT primer and MMLV reverse transcriptase (Promega). PCR was performed in a 50-µl reaction mixture containing 0.25 mM each of the dNTPs, 50 pmol of each primer, and 2.5 units of TAKARA LA Taq (Takara). The resulting PCR amplicons were run on 2% agarose gels with a 1-kb ladder and visualised using UV fluorescence. qRT-PCR amplifications were conducted using an MX3000P Spectrofluorometric Thermal cycler (Stratagene) and RealMasterMix (SYBR Green) kit (Tiangen), initiated with a 2 min incubation at 95°C, followed by 40 cycles of 95°C for 20 s; 58°C for 20 s; 68°C for 20 s. The relative RNA expression levels were normalized by β-actin and measured using a standard curve method [15]. The RT-PCR fragments were cloned with the pGEM-T Easy Vector System (Promega), and sequenced with an ABI PRISM 3730 automated sequencer (Applied Biosystems). The primers used for this study are included in Table S3.

## Supporting Information

Figure S1 **Length distribution for the assembled retroelements in the** ***L. migratoria*** **transcriptome.**
(EPS)

Figure S2 **Protein domains in the seven full-length retroelements identified in this study.** The rectangles in light gray indicate *gag* or *pol* proteins.
(EPS)

Figure S3 **Validation of RNA-seq based expression profiles by qRT-PCR.** The relative transcriptional levels for 10 genes were determined by real-time qRT-PCR using cDNA as template.
(EPS)

Figure S4 **Boxplot showing the transcriptional activity of retroelemnts in different stages.** The staple line for the egg stage is not shown.
(EPS)

Table S1 **Random validation of transcriptome assemblies by cloning and Sanger sequencing of RT-PCR products.**
(XLS)

Table S2 **Control gene sets used to compare with the transcriptional activities of retroelements.**
(XLS)

Table S3 **Primer sequences used for RT-PCR validation experiments.**
(XLS)
